# Research progress on the influence of tumor microenvironment on immunotherapy for pancreatic ductal adenocarcinoma

**DOI:** 10.1515/med-2025-1323

**Published:** 2026-01-26

**Authors:** Yuntao Yang, Yuying Zhang, Zhengchao Shen, Suhang Chen, Yajing Zhang, Xiaoming Wang

**Affiliations:** Department of Hepatobiliary Surgery, The First Afliated Hospital of Wannan Medical College, Wuhu, Anhui, China; Anhui University of Science and Technology, Huainan, Anhui, China

**Keywords:** combination therapy, immunotherapy, pancreatic cancer, pancreatic ductal adenocarcinoma, tumor microenvironment

## Abstract

Pancreatic ductal adenocarcinoma (PDAC) is a highly aggressive malignancy associated with a poor prognosis and considerable resistance to conventional therapies. While radical surgery may offer benefit for a subset of patients with early-stage disease, recent decades have witnessed notable progress in immunotherapy, yielding encouraging outcomes across both hematologic cancers and solid tumors in preclinical and clinical settings. Despite these advances, PDAC remains largely refractory to current immunotherapeutic strategies, owing largely to its unique tumor microenvironment (TME). The TME plays a pivotal role in modulating tumor progression, metastatic dissemination, and treatment response. It is commonly marked by a profoundly immunosuppressive milieu that attenuates effective anti-tumor immunity and complicates therapeutic intervention. The complex cellular and molecular composition of the TME poses significant challenges for the development of novel treatment modalities. Consequently, there is a growing imperative to therapeutically “reprogram” various components and functions within the TME to improve clinical outcomes in PDAC patients. This review seeks to elucidate how the PDAC TME and its key immunosuppressive constituents influence disease progression and response to immunotherapy. A deeper understanding of these interactions may open avenues for innovative treatment approaches capable of overcoming the barriers imposed by the TME in pancreatic cancer.

## Introduction

Pancreatic cancer ranks among the deadliest cancers, exhibiting a notably high mortality rate. Approximately 90 % of these cases are classified as PDAC, which is recognized as the most aggressive variant. A large proportion of PDAC patients are diagnosed at an advanced stage, leading to a bleak 5-year survival rate of only 11 % [1]. The early signs of this disease are often subtle, leading to rapid progression and a generally poor prognosis. At present, the primary treatments for PDAC are surgery and chemotherapy, though their effectiveness remains limited.

In recent years, immunotherapy has gained recognition as a promising approach for cancer treatment, showing notable progress across various cancer types. However, its efficacy in pancreatic cancer has been mostly limited. This highlights the urgent need to explore the factors contributing to the limited success of immunotherapy in PDAC. Research indicates that a key factor is the tumor’s deep integration within a distinctive TME, where ongoing interactions among various cell types facilitate tumor progression [2], 3]. The TME has become increasingly acknowledged as a critical element that affects both tumor growth and response to therapy. In the sections that follow, we will examine how the TME and its predominant immunosuppressive elements play a role in the advancement of pancreatic cancer and the hurdles faced by immunotherapy.

## Definition and composition of TME

**Figure 1:**
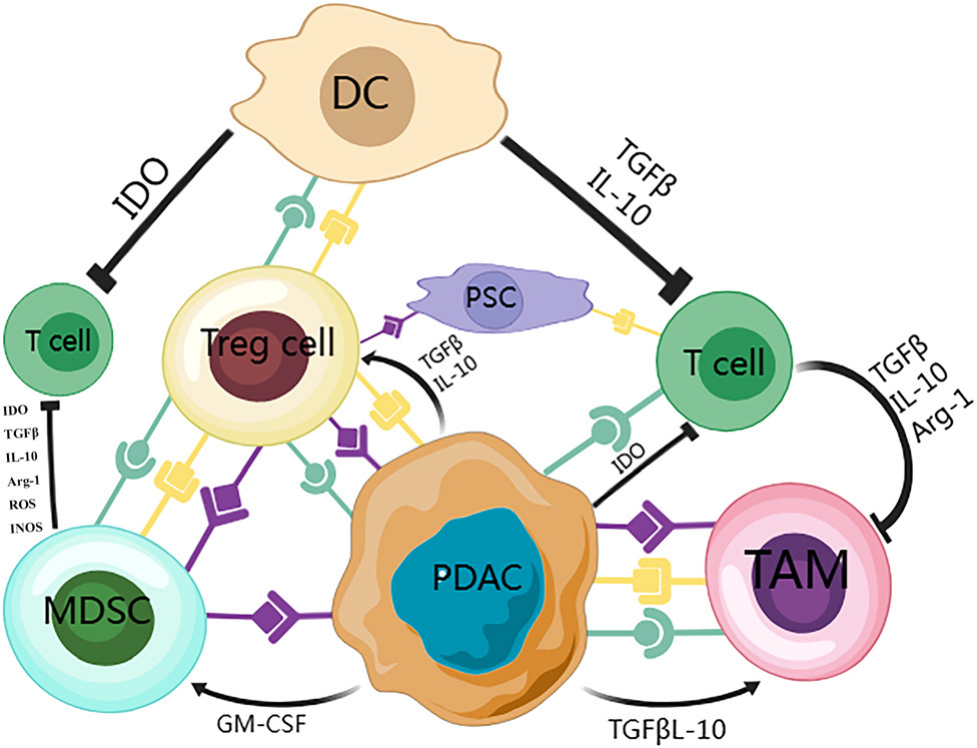
Molecular interactions among different cells within the TME play a crucial role in PDAC. Cancer cells in PDAC employ various mechanisms, including cell surface molecules and soluble factors, to recruit and activate immune-suppressive cells, which directly or indirectly inhibit anti-tumor effector cells, thereby creating an immunosuppressive TME. These suppressive cells can impair effector cell function through mechanisms such as nutrient depletion, phenotypic changes, apoptosis, and energy regulation. Tregs are thought to play a central role in establishing the immunosuppressive TME in PDAC, as they promote the development of tumor-specific immune tolerance.

TME refers to the surrounding area of tumor cells, which plays a critical role in tumor initiation, growth, metastasis, and response to treatment. It encompasses not only the structural, functional, and metabolic components of the tissue where the tumor resides, but also the internal environment of the tumor itself. The TME typically includes various immune cells, such as T lymphocytes, regulatory T cells (Tregs), tumor-associated macrophages (TAMs), and myeloid-derived suppressor cells (MDSCs). Additionally, it consists of the extracellular matrix (ECM) and several secreted molecules, including growth factors, cytokines, and chemokines. Stromal cells, especially cancer-associated fibroblasts (CAFs), are also key elements of this environment (Figure 1). These components often create an immunosuppressive environment that not only impedes normal mechanisms for tumor elimination but also enhances resistance to therapies.

While the composition of the TME shows distinct variations, the immunosuppressive features and mechanisms that shape the diverse immune landscapes in PDAC have not been thoroughly investigated. Current research indicates that different ratios of stromal and immune cell types are associated with varying prognoses for patients with PDAC. In the sections that follow, we will examine the key factors within the TME that influence the effectiveness of tumor immunotherapy.

### Immune cells

In numerous tumors, immune cells make up the largest portion of non-tumor components in the TME. The progression of PDAC is closely linked to various immunosuppressive cell types, such as Tregs, MDSCs, and TAMs, contributing to a highly immunosuppressive TME. Inflammatory responses primarily driven by myeloid cells, such as TAMs and MDSCs, are commonly seen in patients with PDAC. TAMs arise from either infiltrating monocytes or resident tissue macrophages [4]. These cells display considerable plasticity and can be found across a range of polarized states.

#### TAMs

Indeed, immune cells constitute one of the most pivotal stromal elements driving pancreatic ductal adenocarcinoma (PDAC) progression. Hijacking of the immune system is now recognized as a central route through which PDAC acquires therapeutic resistance [5]. Macrophages – key sentinels of innate immunity – dominate the immune landscape of PDAC, where they are commonly termed TAMs [6]. Derived from circulating monocytes, TAMs represent the principal inflammatory population in solid tumors and act as crucial mediators linking chronic inflammation to cancer development [7], [8], [9], [10]. TAMs influence cancer development through multiple pathways, such as initiating tumors via inflammation and supporting tumor progression and metastasis through immune evasion, angiogenesis, cancer cell invasion, and immunosuppression [11], 12]. In addition, TAMs exert potent immunosuppressive effects that shield tumor cells from natural killer (NK) cell- and T lymphocyte-mediated cytotoxicity, both during spontaneous tumor progression and following chemotherapy or immunotherapy.

TAMs, based on *in vitro* research, can be classified into two distinct polarization extremes. M1-like TAMs are believed to have antitumor functions, acting as antigen-presenting cells that produce IL-12, TNF, and inducible nitric oxide synthase. In contrast, M2-like TAMs promote tumor growth and possess immunosuppressive characteristics [13], [14], [15], [16]. Recent single-cell sequencing studies reveal that TAMs can account for 30–50 % of all immune cells in PDAC, predominantly display an M2-polarized phenotype, and are significantly associated with shorter overall survival [17]. Given the limited efficacy of conventional therapies that directly target cancer cells, TAMs have emerged as highly attractive targets for the prevention and treatment of pancreatic cancer.

#### MDSCs

The second key type of immunosuppressive cell in PDAC is MDSC. MDSCs are a group of cells with strong immunosuppressive properties, found in the spleens and tumor tissues of tumor-bearing mice, as well as in the blood and tumor sites of cancer patients [18]. Studies have shown that a network of immune cells, cytokines/chemokines, and signaling pathways critically governs the immunosuppressive activity of MDSCs [19]. In PDAC models, MDSCs mediate potent immune suppression and foster therapeutic resistance by dampening anti-tumor immune responses [20], [21], [22]. Consequently, MDSC frequency can serve as a liquid-biopsy biomarker for tracking disease progression and for predicting responsiveness to immunotherapy or chemotherapy in PDAC patients. Targeting MDSCs is therefore a promising strategy to potentiate both conventional cytotoxic drugs and immune-based agents. Indeed, the number of MDSCs in the peripheral circulation of PDAC patients correlates with advanced tumor stage [23]. Normally, bone marrow cells differentiate from hematopoietic stem cells (HSCs) into various mature cells like macrophages, dendritic cells (DCs), and granulocytes [24]. Novel therapeutic strategies are therefore being designed to elucidate the precise functional mechanisms underlying MDSC-mediated immunosuppression, with the ultimate goal of delivering a breakthrough for PDAC treatment.

#### Tregs

Tregs play a crucial role in balancing autoimmunity and immunosuppression. Within TME, they play a vital role, particularly in suppressing T cell activation. Increasing evidence shows that Tregs not only regulate abnormal immune responses against self-antigens but also significantly impair antitumor immunity and contribute to tumor development [25]. High Treg infiltration in the TME is associated with poor prognosis in multiple malignancies, including PDAC [26], 27]. In patients with PDAC, regulatory Tregs suppress anti-tumour immunity by crippling the activity of CD4^+^ and CD8^+^ T cells, macrophages, NKs and DCs [28], 29]. An expanded Treg pool in the PDAC TME also correlates with metastasis and shorter survival [30]. Yet across cancers, high intratumoural Treg numbers do not always portend a dismal prognosis. Selectively targeting Tregs to restore a pro-inflammatory, immunogenic TME is therefore an increasingly attractive therapeutic concept that has gained considerable momentum in recent years. Systemic ablation of Treg-mediated suppression, however, is constrained by the risk of severe autoimmunity that follows global Treg depletion. A deeper understanding of the molecular underpinnings of Treg inhibition – together with the metabolic reprogramming of both tumour and immune cells – will be essential for the development of safe, precision Treg-directed anti-cancer immunotherapies.

### ECM

ECM is a dense meshwork of structural proteins, adhesive glycoproteins, proteoglycans and enzymes that provides both biochemical cues and mechanical integrity for tissue homeostasis. In PDAC, ECM deposition is markedly increased [31]. Both primary tumours and metastatic lesions display prominent desmoplasia and high abundance of ECM constituents such as hyaluronan and fibrillar collagens [32], indicating that robust fibrosis is a central driver of PDAC pathogenesis and prompting efforts to target ECM components therapeutically. Indeed, specific ECM molecules are linked to patient outcome: high intratumoural hyaluronan expression is associated with a median overall survival of 9.3 months compared with 24.3 months for low-expression tumours [32], whereas another study reported 6.4 vs. 14.6 months for high vs. low type I collagen levels [32]. In addition, aberrant ECM accumulation undermines therapeutic efficacy by acting as a physical barrier that impedes drug delivery while simultaneously activating integrin–focal adhesion kinase (FAK) signaling, which suppresses apoptosis, bolsters pro-survival pathways, and drives chemoresistance [33]. FAK activation is tightly linked to increased ECM stiffness in PDAC [34], and FAK1 itself has been identified as a key driver of both fibrosis and the immunosuppressive TME [35]. Indeed, the majority of human PDAC epithelia exhibit pronounced FAK expression and phosphorylation, a feature almost absent in normal pancreatic epithelium [36]. In KPC mice, treatment with a small-molecule FAK inhibitor markedly reduced tumor fibrosis, progression, and metastasis while concurrently decreasing infiltration of immunosuppressive myeloid cells, ultimately prolonging survival [35].

In summary, the ECM is a fundamental constituent of the pancreatic cancer stroma and plays a pivotal role in disease initiation and clinical progression. Regrettably, despite encouraging pre-clinical data, the most advanced ECM-targeting agent – PEGPH20 – failed to meet its primary end-point in PDAC clinical trials. Although this setback was disappointing, the ECM remains an attractive reservoir of therapeutic targets, and multiple follow-up studies rooted in positive pre-clinical PDAC models are now under way.

### CAFs

CAFs are among the most crucial stromal cells within the TME. Activated CAFs play a role in promoting tumor progression, angiogenesis, invasion, metastasis, and even chemotherapy resistance, often through processes related to ECM remodeling. As key stromal components, CAFs display significant biological heterogeneity in terms of function, phenotype, and origin [37], 38]. In the pancreatic TME, various CAF subsets perform distinct roles [39], 40].

Although most CAF subsets promote tumor progression, some evidence suggests that certain subsets might have the opposite effect [41]. Numerous studies indicate that CAFs are involved in nearly every stage of tumor development via different pathways [42], 43]. By engaging in bidirectional communication with tumor cells and other components of the TME, and by secreting cytokines, chemokines, growth factors, and exosomes, CAFs not only promote tumor growth but also aid cancer cells in evading the immune system [44], 45]. Additionally, CAFs contribute to ECM degradation through the release of matrix metalloproteinases (MMPs) while simultaneously producing new matrix proteins, which supports tumor invasion and angiogenesis [46], 47].

Although therapies aimed at targeting CAFs need careful evaluation to ensure that they focus on tumor-promoting activities, they hold promise for enhancing PDAC treatment when combined with standard chemotherapy. In conclusion, while CAF-targeted therapies should be approached with caution, they offer a promising path to improving current PDAC treatments. Further research is essential to better understand the specific roles and mechanisms of CAFs in the progression of PDAC.

## Immunotherapy for pancreatic cancer and its relationship with the TME

Pancreatic cancer is a highly aggressive malignancy with high rates of morbidity and mortality. Although immunotherapy has proven highly effective in many other cancers, it remains a relatively recent treatment option for pancreatic cancer, used alongside surgery and chemotherapy [48], [49], [50], [51], [52]. Treating PDAC remains a significant challenge, as standard treatments like FOLFIRINOX or gemcitabine-based chemotherapy provide only modest survival benefits, with most patients eventually experiencing disease progression and succumbing to it [53], 54]. One of the key characteristics of PDAC is its immunosuppressive microenvironment, which makes immunotherapy an attractive strategy. However, its effectiveness in pancreatic cancer is limited due to the unique biological behavior and TME of PDAC [55].

The pancreatic TME is highly heterogeneous, posing significant obstacles to immunotherapy. Multiple studies have shown that immune checkpoint inhibitors have minimal efficacy, and although early-phase clinical trials have shown some promise, whole-cell therapeutic vaccines have not succeeded in later-stage trials [48], 56], 57].

**Figure 2:**
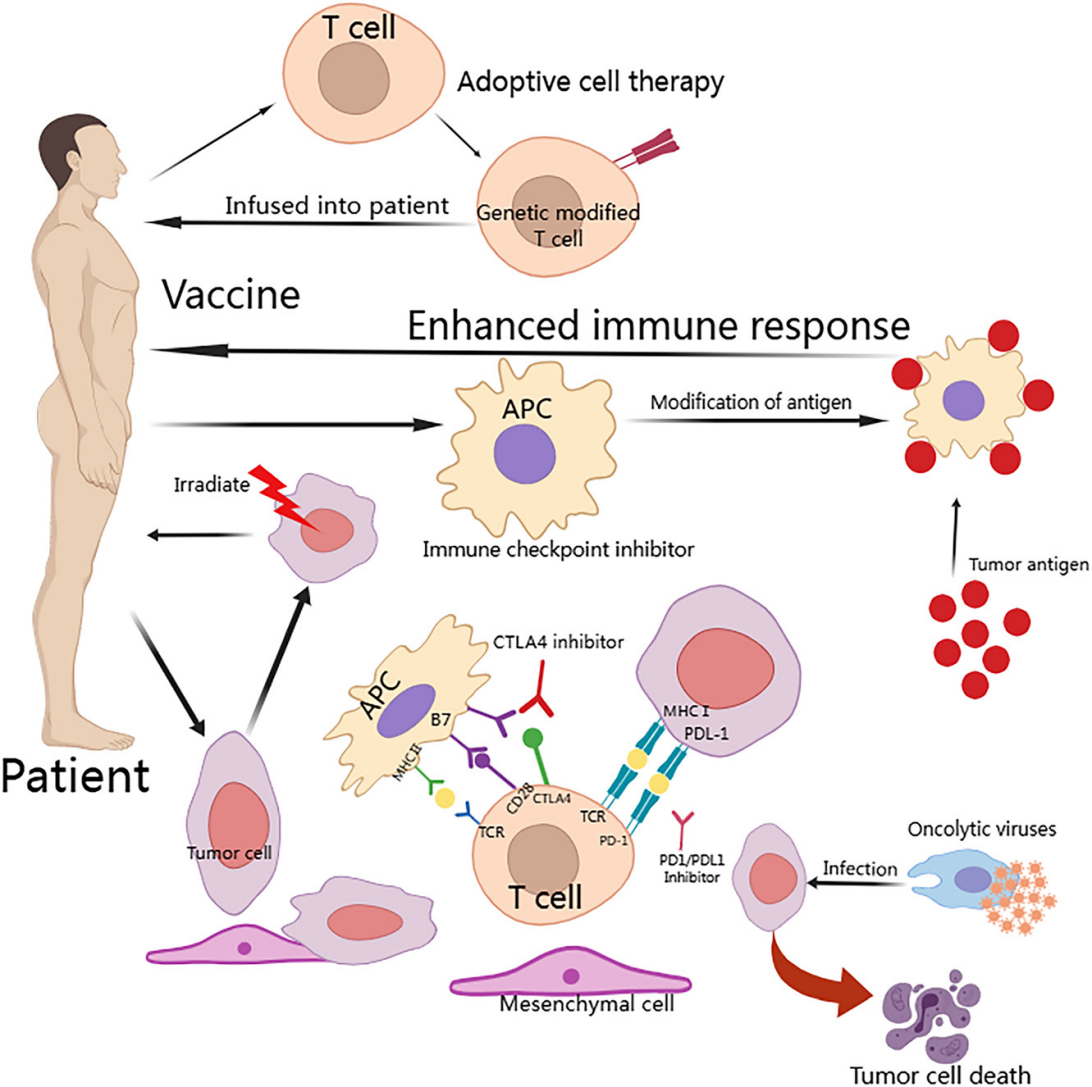
A schematic representation of immunotherapy. The approaches to immune treatment encompass immune checkpoint inhibitors, vaccines, adoptive T cell therapies, oncolytic viruses, and more.

Several factors contribute to these failures, with the TME being a major one, characterized by poor infiltration of effector T cells, prominent myeloid-driven inflammation [57], [58], [59], [60], and a low mutation burden, leading to a scarcity of tumor-associated antigens [61], 62]. However, a small subset of PDAC patients has shown high infiltration of effector T cells and improved overall survival [63], [64], [65], indicating that immunotherapy could be effective in specific cases. This review explores different immunotherapeutic approaches for pancreatic cancer, such as immune checkpoint inhibitors (ICIs), vaccines, adoptive T cell therapies (like CAR-T), and immunomodulatory agents (Figure 2) (Table 1).

**Table 1:** A summary of immunotherapy in pancreatic cancer.

Immunotherapy treatment method	Description	Applicability	Major drugs/Technologies
Immune checkpoint inhibitors	By removing the negative regulatory molecules on T cells, the killing ability of T cells to tumors is enhanced	It is suitable for pancreatic cancer patients expressing the corresponding immune checkpoint molecules	Pembrolizumab nivolumab Ipilimumab tremelimumab
Oncology vaccines	Fights tumors by activating the body’s immune response	It is mainly used for preventive immunization and is used in specific populations	Direct injection of tumor vaccines and genetically engineered vaccines
CAR-T cell therapy	The patient’s own T cells are genetically modified to give them the ability to recognize and destroy tumors	It is mainly for advanced, recurrent or unresectable locally advanced pancreatic cancer	CAR-T cell therapy technology
Adoptive T cell therapy	T Cells are harvested from the patient, expanded and modified *in vitro*, and then infused back into the patient	It can be used as an adjunct to improve the prognosis of patients with certain high-risk factors	Adoptive T cell therapy techniques
Immunomodulators	Cancer control is achieved by influencing the body’s immune microenvironment, such as regulating inflammatory responses or promoting immune cell activation	May be effective in patients with pancreatic cancer who have abnormalities in the immune microenvironment	Interferon a,y–interferon, etc

### Monotherapy immunotherapy for pancreatic cancer

#### ICIs

Cancer immunotherapy has delivered striking efficacy across multiple solid tumours, a leap largely driven by the advent of ICIs that elicit robust anti-tumour activity in most malignancies [66], [67], [68], [69], [70], [71]. These agents are monoclonal antibodies that neutralize key negative regulators of immunity – cytotoxic T-lymphocyte-associated antigen-4 (CTLA-4), programmed cell-death protein-1 (PD-1) and its ligand PD-L1 – thereby dismantling the molecular shields tumours use to evade immune surveillance [72]. By blocking inhibitory receptor–ligand interactions on T cells, ICIs re-invigorate exhausted lymphocytes and amplify physiological anti-tumour responses [73], 74]. Yet this success has not been universal. Despite remarkable advances in nearly every other cancer type, immunotherapy – ICIs in particular – has encountered formidable resistance in pancreatic ductal adenocarcinoma PDAC [56], 75]. Numerous clinical trials testing ICIs or alternative immunotherapeutic strategies in PDAC have yielded largely disappointing outcomes, especially when contrasted with the dramatic responses seen in melanoma, lung, or renal cancers.

Single-cell sequencing of anti-PD-1-treated PDAC mice revealed a surge in IFN-γ and consequent PD-L1 re-expression by day 7 [76]. In a clinical cohort, peripheral Treg frequency rose 3 weeks after pembrolizumab initiation and correlated with early progressive disease [77]. Moreover, in patients who received combined STING agonist plus anti-PD-1 therapy, those developing acquired resistance showed marked intra-tumoral up-regulation of AXL [78].

PDAC is characterized by an “immune-excluded” phenotype in which CD8^+^ T cells are confined to the tumor periphery, rendering the lesion largely refractory to immune-checkpoint blockade. Recent single-cell analyses have begun to dissect the multilayered circuits that orchestrate this exclusion. Tumor-cell-overexpressed DDR1, for example, aligns collagen fibers parallel to the tumor border, erecting a physical wall that impedes CD8^+^ T-cell infiltration; in DDR1-null models, collagen architecture is relaxed, T-cell influx increases and sensitivity to ICI is restored [79]. Cancer-associated fibroblasts further amplify exclusion by secreting TGF-β, which activates the CXCL12-CXCR4 axis and sequesters T cells in the stroma; blockade of TGF-β reverses this process and synergizes with anti-PD-1 therapy [80]. Mechanically, elevated ECM stiffness triggers nuclear translocation of focal-adhesion kinase (FAK), and the resultant STAT1/STAT3 complex represses MHC-I expression, blunting tumor recognition by T cells; FAK inhibitors re-establish antigen presentation and augment ICI efficacy [81]. Finally, single-cell profiling has revealed a sex-specific pathway: FPR2^+^ macrophages are markedly expanded in female PDAC and, via CCL2-and TGF-β-rich secretomes, intensify collagen deposition and T-cell exclusion; pharmacological antagonism of FPR2 enhances anti-PD-1 activity in pre-clinical models [82].

Collectively, these high-resolution studies portray PDAC immune exclusion as a multifactorial process – involving collagen re-organization, aberrant signaling, mechanotransduction and sex-biased immune circuits – and provide a rational framework for designing combination regimens that convert immune-desert tumors into immune-responsive lesions.

#### Tumor vaccines

Tumor vaccines have demonstrated the ability to elicit robust immune responses against tumors, but their performance in early-stage pancreatic cancer has been underwhelming. These vaccines, which consist of whole tumor cells, peptides, proteins, and recombinant constructs, aim to stimulate circulating tumor-specific T cells to target and destroy cancerous cells. Some research has also investigated the effectiveness of therapeutic vaccines in PDAC, often in combination with ICIs or chemotherapy. Among these, GVAX is the most studied [83]. GVAX is a whole-cell vaccine system commonly used for treating various cancers, including PDAC (84). The PDAC-specific version of GVAX is derived from two pancreatic cancer cell lines that have been genetically engineered to express GM-CSF. The vaccines are given intradermally and release significant amounts of GM-CSF, which draws in antigen-presenting cells (APCs) and promotes their maturation. While the vaccine has proven safe, its clinical impact has been limited [83], 84].

It is important to note that GM-CSF alone is insufficient for full APC maturation, as the presence of IL-4 is also required. The GVAX vaccine, which involves allogeneic pancreatic cancer cells modified to produce GM-CSF, has sometimes been paired with cyclophosphamide to deplete the TME and has shown the ability to induce T cell infiltration into the PDAC microenvironment [85], 86]. In small-scale phase I trials, almost all such vaccines produced tumor-specific T cell immunity in some patients [87], 88]. Interestingly, patients who developed vaccine-specific T cell responses in early trials often had higher survival rates. However, these early results have not been replicated in larger trials. A phase III trial using a single peptide derived from human telomerase (hTERT) failed to show a survival benefit for patients with metastatic disease, even among those who had an immune response [89]. Whole-cell vaccines have the potential to enhance the immune response to both tumor-specific and self-antigens, but their overall effectiveness has been constrained. GVAX, which uses irradiated allogeneic PDAC cells expressing GM-CSF, did not improve survival in metastatic PDAC in a phase IIb/III trial, even among immune responders [90]. These outcomes have lessened enthusiasm for therapeutic vaccines (Table 2).

**Table 2:** An overview of tumor vaccine therapy in pancreatic cancer.

Vaccine name (type)	Clinical phase	Treatment regimen	Key outcomes	References
Autogene cevumeran (BNT122) (individualized mRNA neoantigen vaccine)	Phase 1	Surgery + anti-PD-L1 (atezolizumab) + vaccine + mFOLFIRINOX chemotherapy	−50 % (8/16) of patients mounted a T-cell response − Median RFS not reached (>3.2 years) in responders vs. 13.4 months in non-responders −86 % reduced risk of recurrence at 3 years in responders	[114]
ELI-002 2 P (off-the-shelf KRAS-targeted mRNA vaccine)	Phase 1	Vaccine monotherapy (targeting KRAS mutations)	−84 % (21/25) T-cell response rate − mOS of 28.9 months in pancreatic patients, mRFS>15 months − Stronger T-cell responses associated with longer survival	[115]
GVAX ± CRS-207 (whole-cell vaccine + bacterial vaccine)	Phase 2	GVAX sequentially with CRS-207 vs. GVAX alone	− mOS 6.1 months in combo vs. 3.9 months with GVAX alone (HR=0.54) − In patients receiving≥3 doses: mOS 9.7 months (combo) vs. 4.6 months (GVAX alone) (HR=0.44)	[116]
GVAX + nivolumab±urelumab (whole-cell vaccine + ICIs/agonist)	Phase 1/2	Neoadjuvant/adjuvant setting. Three arms: A: GVAX; B: GVAX+Nivo; C: GVAX+Nivo+Urelumab	−30 % (3/10) achieved pathologic response (all partial) −9 patients remained disease-free at median 12-month follow-up − Tolerable toxicity, no severe irAEs	[117]

#### Adoptive T cell therapy

Adoptive cell therapy is another form of immunotherapy, commonly applied to hematological cancers but increasingly being tested for solid tumors. T cell therapy, which involves the adoptive transfer of genetically modified tumor-targeting T cells, is emerging as a promising treatment for challenging cancers like PDAC [91]. Two primary methods are used for genetic modification [1]: T cells are engineered to express T cell receptors (TCRs) that recognize tumor antigens in the context of human leukocyte antigen (HLA); or [2] T cells are engineered to express chimeric antigen receptors (CARs), which directly bind to proteins, carbohydrates, or glycolipids on cancer cell surfaces, allowing them to bypass the frequent HLA downregulation observed in solid tumors [92].

CAR-T cell therapy has demonstrated remarkable success in specific forms of B-cell leukemia and lymphoma, but it encounters substantial obstacles that restrict its wider use in solid tumors and other hematological malignancies. In blood cancers, studies have shown that lymphodepletion before CAR-T cell therapy can improve efficacy, but the role of such conditioning regimens in solid tumors remains unclear [93].

CAR-T cells function as a “living drug,” customized for each individual patient. Present treatments involve harvesting the patient’s T cells, altering them genetically to produce CARs that detect and attach to particular proteins (tumor antigens) found on the surfaces of cancer cells. While cyclophosphamide, alone or combined with fludarabine (Cy/Flu), is the most common chemotherapy used in CAR-T trials, these drugs are not typically used for PDAC. Whether these or other cytotoxic therapies commonly used for PDAC, such as gemcitabine- or fluoropyrimidine-based treatments, can improve CAR-T efficacy is still being studied. These treatments may modify the tumor microenvironment (TME) by inducing immunogenic cell death, promoting local T cell infiltration, Tregs and MDSCs [94].

Despite the potential, several hurdles impede the success of CAR-T therapy, including serious toxicities like cytokine release syndrome (CRS), insufficient anti-tumor efficacy, antigen escape, and limited T cell trafficking [95], 96].

#### Immunomodulator

In pancreatic cancer, tumor cells release immunosuppressive cytokines that shape the TME, aiding in immune evasion. Immunomodulators are non-specific biological agents that enhance, promote, and regulate immune function. In pancreatic cancer treatment, commonly used immunomodulators include interferons (IFNs), interleukins (ILs), and other related molecules. Research has demonstrated that combining IL-10 with oncolytic viruses based on the vaccinia virus can boost the immune response against pancreatic cancer cells. IL-10, however, inhibits the secretion of IFN-γ and granzyme B, reducing the antitumor activity of CAR-T cells. Depleting IL-10 in the TME can significantly restore CAR-T cell function [97].

TGF-β plays a role in inducing T cells to acquire a regulatory phenotype. Blocking TGF-β signaling can reduce the activity of regulatory T cells in pancreatic cancer, thereby enhancing anti-tumor immunity [98]. Furthermore, combining IL-6 inhibitors with anti-PD-1 therapy has been shown to increase the number of CD8+ T cells within tumors, improving antitumor effectiveness compared to using immune checkpoint inhibitors alone [99].

### Combination immunotherapy for pancreatic cancer

In solid tumors, low tumor immunogenicity and a highly immunosuppressive TME contribute to the intrinsic resistance to immune checkpoint blockade (ICB) therapy [100]. While immune checkpoint inhibitors (ICIs) can block inhibitory pathways that suppress effector T cell activity, the TME in pancreatic cancer contains numerous soluble factors that hinder the function of these effector T cells. Pancreatic cancer is characterized by a dense stroma, a low presence of tumor-infiltrating lymphocytes (TILs), and its classification as a “cold tumor,” which diminishes the efficacy of certain ICIs. The thick fibrotic matrix surrounding pancreatic cancer cells not only acts as a physical barrier but also limits drug penetration [101]. Reduced effector T cell presence and increased immunosuppressive cells within the TME contribute to creating a strongly immunosuppressive environment in pancreatic cancer, making ICI therapy alone largely ineffective [5]. Additionally, the fibrotic matrix may prevent TILs from infiltrating the tumor, while high expression of inhibitory receptors or ligands leads to T cell exhaustion. Immunosuppressive cells within the TME can further impair CD8+ T cell function, either through direct contact or paracrine signaling, contributing to resistance to ICIs [102]. Patients with low neoantigen heterogeneity and a higher number of clonal neoantigens tend to respond better to ICIs. However, given the low mutation burden in pancreatic cancer, the response to ICIs remains poor [103].

Although single-agent immunotherapy has not delivered the desired results, combining it with other treatment modalities has shown potential to significantly improve outcomes. Whole-genome analyses have revealed significant heterogeneity among pancreatic cancer patients, suggesting that personalized treatment approaches may enhance the effectiveness of immunotherapy. Given the variability in individual responses, some studies have developed an “immune score,” combining immunohistochemistry and gene expression data to evaluate immune cell infiltration and predict immunotherapy efficacy [104]. The role of the TME in pancreatic cancer metastasis highlights the importance of understanding its unique features in designing more effective immunotherapeutic strategies [50].

Looking ahead, future immunotherapies for pancreatic cancer are likely to shift from single-agent ICIs to combination therapies. This could include combinations of different immunotherapies or integrating immunotherapy with chemotherapy, radiotherapy, and targeted therapy [105], 106]. The success of immunotherapy depends on activating or modulating the immune system to specifically target tumor cells, and various immunotherapeutic approaches can achieve this [107] (Table 3).

**Table 3:** An overview of immune checkpoint inhibitor therapy in pancreatic cancer.

Trial name/Description	Treatment regimen	Phase	Primary outcomes	References
ICI + chemotherapy	Nivolumab + chemotherapy	Phase II	1-year OS rate of 57.7 % (vs. 35 % in historical chemotherapy control)	[118]
Meta-analysis: ICI + chemotherapy	ICI + chemotherapy vs. Chemotherapy alone	Meta-analysis	Improved OS (HR=0.82; 95 % CI: 0.78–0.87)	[119]
Retrospective study in MSI-H/dMMR PDAC	Anti-PD-1 ± anti-CTLA-4	Multicenter retrospective	ORR 48.4 %, median PFS 26.7 months, median OS not reached	[120]
Meta-analysis: ICI + radiotherapy	ICI + radiotherapy vs. Control	Meta-analysis	Potential increased risk of death (HR=1.18; 95 % CI: 1.04–1.34)	[121]

Finding the most effective combination therapies will be essential for progressing immunotherapy in pancreatic cancer. While this review focuses on immunotherapy, key combinations will likely involve standard treatments like chemotherapy and radiotherapy, which can enhance the immune response through immunogenic cell death. The vast number of potential combinations exceeds the current capacity for clinical trials, so those targeting complementary mechanisms in the anti-tumor immune response will be prioritized. For instance, integrating therapeutic vaccines to stimulate T cells, checkpoint inhibitors to avoid exhaustion, and matrix modulation to improve T cell infiltration could address different mechanisms and may prove to be highly effective. However, challenges remain, including managing toxicity, optimizing dosage, and determining the best treatment sequence, especially for immune agonists. For instance, while the combination of nivolumab and ipilimumab has significantly improved survival in melanoma patients compared to monotherapy, it also increases toxicity and cost [108], [109], [110], [111].

An ideal combination therapy would also include reliable immune pharmacodynamic biomarkers to quickly assess treatment response. Because of the heterogeneity of tumors and individual differences, pancreatic cancer has exhibited a lower response rate to immunotherapy in comparison to other solid tumors. However, based on results from other cancers, combining immunotherapy with chemoradiotherapy and targeted therapies is expected to outperform single-agent treatments [90], 112]. These combination therapies can potentially improve survival by reshaping the immune microenvironment and converting “cold” tumors into “hot” ones [113]. Continued research into diverse immunotherapy approaches and rational combinations with other treatments may offer the most promising outcomes for PDAC.

Due to the heterogeneity of tumors and individual differences in pancreatic cancer, immunotherapy has not proven to be as effective as it is for other solid tumors. However, based on promising results from other cancers, combining immunotherapy with chemoradiotherapy and targeted therapies is expected to be more effective than single-agent approaches [90], 112]. With many possible combinations and a relatively small patient population, prioritizing treatments targeting distinct mechanisms supported by preclinical studies will be essential. Although treating PDAC is challenging, there is still hope for the development of effective therapies that can treat and eventually prevent this devastating disease.

## Conclusion and prospect

The treatment landscape for PDAC is shifting, as therapies have historically been based on patient functional status and disease stage. In 2023, we are experiencing a time of therapeutic progress and heightened complexity, with a stronger focus on identifying biomarkers for categorizing patient subgroups. Nevertheless, there is still a demand for more reliable predictive and prognostic biomarkers to inform treatment choices. As clinical trials and technologies evolve over the next decade, outcomes for PDAC are expected to improve with the broader adoption of biomarker-driven strategies for cytotoxic chemotherapy, targeted therapies, and immunotherapy.

In the past decade, preclinical and clinical studies have highlighted the importance of both the adaptive and innate immune systems in the immune surveillance of PDAC. Yet, clinical results from PDAC immunotherapy trials have been less promising. It is critical to reconcile these encouraging preclinical findings with the underwhelming clinical trial outcomes and to understand why certain trials fail despite sound scientific foundations. This review focuses on the characteristics of the TME in PDAC and its influence on immunotherapy. Emerging evidence highlights the need to understand the complex roles that TME components play in both tumor suppression and progression, making it essential to study the drivers of TME organization and immunosuppression while exploring ways to target them effectively.

Although immunotherapy has shown initial promise in treating pancreatic cancer, achieving substantial clinical benefits has proven challenging. Each failed late-stage trial has highlighted the growing recognition that single-agent immunotherapy is unlikely to succeed in PDAC, making combination therapies a more promising approach. As immunological strategies for treating and managing PDAC continue to evolve, improving patient quality of life should remain a top priority. Many immunotherapy trials for PDAC have yielded disappointing results, largely due to the immunosuppressive nature of the TME. Consequently, existing immunotherapies must be refined and optimized to overcome this barrier, and novel immunotherapy targets identified in preclinical studies must be further explored in human clinical trials to confirm their potential.

One of the key questions yet to be answered is why many pancreatic cancer patients do not respond to immunotherapy. We must also explore the feedback mechanisms that occur during treatment and how they can be leveraged in combination therapies, particularly when factoring in prior chemotherapy and/or radiotherapy. Another important consideration is how to tailor treatment decisions based on the personalized characteristics of each patient’s TME. Given the relatively low incidence of PDAC, future trials should prioritize in-depth analysis of the TME and personalized treatment approaches to accelerate progress toward more effective therapies.

Despite numerous trial setbacks, our growing understanding of the PDAC TME and new therapeutic strategies offer hope for more effective future treatments. In summary, PDAC continues to be a highly deadly disease, underscoring the urgent need for innovative and enhanced treatment options. While immunotherapy has achieved significant success in other solid tumors, its application in PDAC, due to the complex TME, may require combination approaches rather than single-agent therapies. Although identifying the optimal combinations and selecting the right patients remain challenging, carefully designed studies are essential for maximizing the potential of immunotherapy and enhancing outcomes for this challenging disease.

## References

[1] Stoffel EM, Brand RE, Goggins M (2023). Pancreatic cancer: changing epidemiology and new approaches to risk assessment, early detection, and prevention. Gastroenterology.

[2] Cheng K, Cai N, Zhu J, Yang X, Liang H, Zhang W (2022). Tumor-associated macrophages in liver cancer: from mechanisms to therapy. Cancer Commun.

[3] Ho WJ, Jaffee EM, Zheng L (2020). The tumour microenvironment in pancreatic cancer - clinical challenges and opportunities. Nat Rev Clin Oncol.

[4] Zhu Y, Herndon JM, Sojka DK, Kim KW, Knolhoff BL, Zuo C (2017). Tissue-resident macrophages in pancreatic ductal adenocarcinoma originate from embryonic hematopoiesis and promote tumor progression. Immunity.

[5] Sideras K, Braat H, Kwekkeboom J, van Eijck CH, Peppelenbosch MP, Sleijfer S (2014). Role of the immune system in pancreatic cancer progression and immune modulating treatment strategies. Cancer Treat Rev.

[6] Balkwill FR, Mantovani A (2012). Cancer-related inflammation: common themes and therapeutic opportunities. Semin Cancer Biol.

[7] Chen Q, Zhang XH, Massagué J (2011). Macrophage binding to receptor VCAM-1 transmits survival signals in breast cancer cells that invade the lungs. Cancer Cell.

[8] Fan QM, Jing YY, Yu GF, Kou XR, Ye F, Gao L (2014). Tumor-associated macrophages promote cancer stem cell-like properties via transforming growth factor-beta1-induced epithelial-mesenchymal transition in hepatocellular carcinoma. Cancer Lett.

[9] Kurahara H, Takao S, Kuwahata T, Nagai T, Ding Q, Maeda K (2012). Clinical significance of folate receptor β-expressing tumor-associated macrophages in pancreatic cancer. Ann Surg Oncol.

[10] Ségaliny AI, Mohamadi A, Dizier B, Lokajczyk A, Brion R, Lanel R (2015). Interleukin-34 promotes tumor progression and metastatic process in osteosarcoma through induction of angiogenesis and macrophage recruitment. Int J Cancer.

[11] Cassetta L, Pollard JW (2018). Targeting macrophages: therapeutic approaches in cancer. Nat Rev Drug Discov.

[12] Mantovani A, Marchesi F, Malesci A, Laghi L, Allavena P (2017). Tumour-associated macrophages as treatment targets in oncology. Nat Rev Clin Oncol.

[13] Geiger R, Rieckmann JC, Wolf T, Basso C, Feng Y, Fuhrer T (2016). L-Arginine modulates T cell metabolism and enhances survival and anti-tumor activity. Cell.

[14] Propper DJ, Balkwill FR (2022). Harnessing cytokines and chemokines for cancer therapy. Nat Rev Clin Oncol.

[15] Batlle E, Massagué J (2019). Transforming growth Factor-β signaling in immunity and cancer. Immunity.

[16] Peng D, Fu M, Wang M, Wei Y, Wei X (2022). Targeting TGF-β signal transduction for fibrosis and cancer therapy. Mol Cancer.

[17] Yang S, Liu Q, Liao Q (2020). Tumor-associated macrophages in pancreatic ductal adenocarcinoma: origin, polarization, function, and reprogramming. Front Cell Dev Biol.

[18] Vincent A, Herman J, Schulick R, Hruban RH, Goggins M (2011). Pancreatic cancer. Lancet (London, England).

[19] Panni RZ, Sanford DE, Belt BA, Mitchem JB, Worley LA, Goetz BD (2014). Tumor-induced STAT3 activation in monocytic myeloid-derived suppressor cells enhances stemness and mesenchymal properties in human pancreatic cancer. Cancer Immunol Immunother : CII..

[20] Zheng D, Chen H, Bartee MY, Williams J, Davids JA, Lomas DA (2013). Myxomaviral anti-inflammatory serpin reduces myeloid-derived suppressor cells and human pancreatic cancer cell growth in mice. J Cancer Sci Ther.

[21] Lu Z, Long Y, Wang Y, Wang X, Xia C, Li M (2021). Phenylboronic acid modified nanoparticles simultaneously target pancreatic cancer and its metastasis and alleviate immunosuppression. Eur J Pharm Biopharm : off J of Arbeitsgemeinschaft fur Pharmazeutische Verfahrenstechnik eV.

[22] Metzger P, Kirchleitner SV, Boehmer DFR, Hörth C, Eisele A, Ormanns S (2020). Systemic but not MDSC-specific IRF4 deficiency promotes an immunosuppressed tumor microenvironment in a murine pancreatic cancer model. Cancer Immunol Immunother : CII..

[23] Pergamo M, Miller G (2017). Myeloid-derived suppressor cells and their role in pancreatic cancer. Cancer Gene Ther.

[24] Schulz C, Gomez Perdiguero E, Chorro L, Szabo-Rogers H, Cagnard N, Kierdorf K (2012). A lineage of myeloid cells independent of Myb and hematopoietic stem cells. Science (New York, N.Y.).

[25] Vonderheide RH, Bear AS (2020). Tumor-derived myeloid cell chemoattractants and T cell exclusion in pancreatic cancer. Front Immunol.

[26] Huang H, Wang Z, Zhang Y, Pradhan RN, Ganguly D, Chandra R (2022). Mesothelial cell-derived antigen-presenting cancer-associated fibroblasts induce expansion of regulatory T cells in pancreatic cancer. Cancer Cell.

[27] Xu Y, Fu J, Henderson M, Lee F, Jurcak N, Henn A (2023). CLDN18.2 BiTE engages effector and regulatory T cells for antitumor immune response in preclinical models of pancreatic cancer. Gastroenterology.

[28] Zhang J, Meng H, Zhang M, Zhang C, Huang M, Yan C (2020). Regulation of docetaxel chemosensitivity by NR2F6 in breast cancer. Endocr Relat Cancer.

[29] Ohue Y, Nishikawa H (2019). Regulatory T (Treg) cells in cancer: can treg cells be a new therapeutic target?. Cancer Sci.

[30] Whiteside TL (2018). FOXP3+ treg as a therapeutic target for promoting anti-tumor immunity. Expert Opin Ther Targets.

[31] Ohlund D, Lundin C, Ardnor B, Oman M, Naredi P, Sund M (2009). Type IV collagen is a tumour stroma-derived biomarker for pancreas cancer. Br J Cancer.

[32] Whatcott CJ, Diep CH, Jiang P, Watanabe A, LoBello J, Sima C (2015). Desmoplasia in primary tumors and metastatic lesions of pancreatic cancer. Clin Cancer Res : an off j of American Assoc Cancer Res.

[33] Diaz Osterman CJ, Ozmadenci D, Kleinschmidt EG, Taylor KN, Barrie AM, Jiang S (2019). FAK activity sustains intrinsic and acquired ovarian cancer resistance to platinum chemotherapy. eLife.

[34] Stokes JB, Adair SJ, Slack-Davis JK, Walters DM, Tilghman RW, Hershey ED (2011). Inhibition of focal adhesion kinase by PF-562,271 inhibits the growth and metastasis of pancreatic cancer concomitant with altering the tumor microenvironment. Mol Cancer Therapeut.

[35] Jiang H, Hegde S, Knolhoff BL, Zhu Y, Herndon JM, Meyer MA (2016). Targeting focal adhesion kinase renders pancreatic cancers responsive to checkpoint immunotherapy. Nat Med.

[36] Jiang H, Liu X, Knolhoff BL, Hegde S, Lee KB, Jiang H (2020). Development of resistance to FAK inhibition in pancreatic cancer is linked to stromal depletion. Gut.

[37] Liu T, Han C, Wang S, Fang P, Ma Z, Xu L (2019). Cancer-associated fibroblasts: an emerging target of anti-cancer immunotherapy. J Hematol Oncol.

[38] Bu L, Baba H, Yoshida N, Miyake K, Yasuda T, Uchihara T (2019). Biological heterogeneity and versatility of cancer-associated fibroblasts in the tumor microenvironment. Oncogene.

[39] Kalluri R (2016). The biology and function of fibroblasts in cancer. Nat Rev Cancer.

[40] Biffi G, Tuveson DA (2021). Diversity and biology of cancer-associated fibroblasts. Physiol Rev.

[41] Mizutani Y, Kobayashi H, Iida T, Asai N, Masamune A, Hara A (2019). Meflin-positive cancer-associated fibroblasts inhibit pancreatic carcinogenesis. Cancer Res.

[42] Fiori ME, Di Franco S, Villanova L, Bianca P, Stassi G, De Maria R (2019). Cancer-associated fibroblasts as abettors of tumor progression at the crossroads of EMT and therapy resistance. Mol Cancer.

[43] Joshi RS, Kanugula SS, Sudhir S, Pereira MP, Jain S, Aghi MK (2021). The role of cancer-associated fibroblasts in tumor progression. Cancers.

[44] Martinez-Outschoorn UE, Lisanti MP, Sotgia F (2014). Catabolic cancer-associated fibroblasts transfer energy and biomass to anabolic cancer cells, fueling tumor growth. Semin Cancer Biol.

[45] Kobayashi H, Enomoto A, Woods SL, Burt AD, Takahashi M, Worthley DL (2019). Cancer-associated fibroblasts in gastrointestinal cancer. Nat Rev Gastroenterol Hepatol.

[46] Fullár A, Dudás J, Oláh L, Hollósi P, Papp Z, Sobel G (2015). Remodeling of extracellular matrix by normal and tumor-associated fibroblasts promotes cervical cancer progression. BMC Cancer.

[47] Eble JA, Niland S (2019). The extracellular matrix in tumor progression and metastasis. Clin Exp Metastasis.

[48] Gao X, Xu N, Li Z, Shen L, Ji K, Zheng Z (2023). Safety and antitumour activity of cadonilimab, an anti-PD-1/CTLA-4 bispecific antibody, for patients with advanced solid tumours (COMPASSION-03): a multicentre, open-label, phase 1b/2 trial. Lancet Oncol.

[49] Somaiah N, Conley AP, Parra ER, Lin H, Amini B, Solis Soto L (2022). Durvalumab plus tremelimumab in advanced or metastatic soft tissue and bone sarcomas: a single-centre phase 2 trial. Lancet Oncol.

[50] Buisseret L, Loirat D, Aftimos P, Maurer C, Punie K, Debien V (2023). Paclitaxel plus carboplatin and durvalumab with or without oleclumab for women with previously untreated locally advanced or metastatic triple-negative breast cancer: the randomized SYNERGY phase I/II trial. Nat Commun.

[51] Du Y, Dai J, Mao L, Wei X, Bai X, Chen L (2024). Phase Ib study of anlotinib in combination with anti-PD-L1 antibody (TQB2450) in patients with advanced acral melanoma. J Eur Acad Dermatol Venereol : JEADV.

[52] Verschoor YL, van de Haar J, van den Berg JG, van Sandick JW, Kodach LL, van Dieren JM (2024). Neoadjuvant atezolizumab plus chemotherapy in gastric and gastroesophageal junction adenocarcinoma: the phase 2 PANDA trial. Nat Med.

[53] Conroy T, Hammel P, Hebbar M, Ben Abdelghani M, Wei AC, Raoul JL (2018). FOLFIRINOX or gemcitabine as adjuvant therapy for pancreatic cancer. N Engl J Med.

[54] Dhir M, Zenati MS, Hamad A, Singhi AD, Bahary N, Hogg ME (2018). FOLFIRINOX versus Gemcitabine/Nab-paclitaxel for neoadjuvant treatment of resectable and borderline resectable pancreatic head adenocarcinoma. Ann Surg Oncol.

[55] Chandana S, Babiker HM, Mahadevan D (2019). Therapeutic trends in pancreatic ductal adenocarcinoma (PDAC). Expet Opin Invest Drugs.

[56] Royal RE, Levy C, Turner K, Mathur A, Hughes M, Kammula US (2010). Phase 2 trial of single agent ipilimumab (anti-CTLA-4) for locally advanced or metastatic pancreatic adenocarcinoma. J Immunother.

[57] Vonderheide RH, Bayne LJ (2013). Inflammatory networks and immune surveillance of pancreatic carcinoma. Curr Opin Immunol.

[58] Johnson BA, Yarchoan M, Lee V, Laheru DA, Jaffee EM (2017). Strategies for increasing pancreatic tumor immunogenicity. Clin Cancer Res : an off Journal of American Assoc Cancer Res.

[59] Stromnes IM, Hulbert A, Pierce RH, Greenberg PD, Hingorani SR (2017). T-cell localization, activation, and clonal expansion in human pancreatic ductal adenocarcinoma. Cancer Immunol Res.

[60] Curiel TJ, Coukos G, Zou L, Alvarez X, Cheng P, Mottram P (2004). Specific recruitment of regulatory T cells in ovarian carcinoma fosters immune privilege and predicts reduced survival. Nat Med.

[61] Dreyer SB, Chang DK, Bailey P, Biankin AV (2017). Pancreatic cancer genomes: implications for clinical management and therapeutic development. Clin Cancer Res : an official J of American Assoc Cancer Res.

[62] Balli D, Rech AJ, Stanger BZ, Vonderheide RH (2017). Immune cytolytic activity stratifies molecular subsets of human pancreatic cancer. Clin Cancer Res : an off j of American Assoc Cancer Res.

[63] Ino Y, Yamazaki-Itoh R, Shimada K, Iwasaki M, Kosuge T, Kanai Y (2013). Immune cell infiltration as an indicator of the immune microenvironment of pancreatic cancer. Br J Cancer.

[64] Balachandran VP, Łuksza M, Zhao JN, Makarov V, Moral JA, Remark R (2017). Identification of unique neoantigen qualities in long-term survivors of pancreatic cancer. Nature.

[65] Chen DS, Mellman I (2017). Elements of cancer immunity and the cancer-immune set point. Nature.

[66] Hodi FS, O’Day SJ, McDermott DF, Weber RW, Sosman JA, Haanen JB (2010). Improved survival with ipilimumab in patients with metastatic melanoma. N Engl J Med.

[67] Robert C, Thomas L, Bondarenko I, O’Day S, Weber J, Garbe C (2011). Ipilimumab plus dacarbazine for previously untreated metastatic melanoma. N Engl J Med.

[68] Borghaei H, Paz-Ares L, Horn L, Spigel DR, Steins M, Ready NE (2015). Nivolumab versus docetaxel in advanced nonsquamous non-small-cell lung cancer. N Engl J Med.

[69] Larkin J, Chiarion-Sileni V, Gonzalez R, Grob JJ, Cowey CL, Lao CD (2015). Combined nivolumab and ipilimumab or monotherapy in untreated melanoma. N Engl J Med.

[70] Gibney GT, Weiner LM, Atkins MB (2016). Predictive biomarkers for checkpoint inhibitor-based immunotherapy. Lancet Oncol.

[71] Darvin P, Toor SM, Sasidharan Nair V, Elkord E (2018). Immune checkpoint inhibitors: recent progress and potential biomarkers. Exp Mol Med.

[72] Wei SC, Duffy CR, Allison JP (2018). Fundamental mechanisms of immune checkpoint blockade therapy. Cancer Discov.

[73] Kabacaoglu D, Ciecielski KJ, Ruess DA, Algül H (2018). Immune checkpoint inhibition for pancreatic ductal adenocarcinoma: current limitations and future options. Front Immunol.

[74] Stone ML, Beatty GL (2019). Cellular determinants and therapeutic implications of inflammation in pancreatic cancer. Pharmacol Ther.

[75] Brahmer JR, Tykodi SS, Chow LQ, Hwu WJ, Topalian SL, Hwu P (2012). Safety and activity of anti-PD-L1 antibody in patients with advanced cancer. N Engl J Med.

[76] Chen Q, Yin H, Jiang Z, He T, Xie Y, Mao W (2024). Poor clinical outcomes and immunoevasive contexture in CD161(+)CD8(+) T cells barren human pancreatic cancer. J immunotherapy of cancer.

[77] Sivakumar S, Jainarayanan A, Arbe-Barnes E, Sharma PK, Leathlobhair MN, Amin S (2025). Distinct immune cell infiltration patterns in pancreatic ductal adenocarcinoma (PDAC) exhibit divergent immune cell selection and immunosuppressive mechanisms. Nat Commun.

[78] Yang J, Yu X, Xiao M, Xu H, Tan Z, Lei Y (2025). Histone lactylation-driven feedback loop modulates cholesterol-linked immunosuppression in pancreatic cancer. Gut.

[79] Su H, Yang F, Fu R, Trinh B, Sun N, Liu J (2022). Collagenolysis-dependent DDR1 signalling dictates pancreatic cancer outcome. Nature.

[80] Mariathasan S, Turley SJ, Nickles D, Castiglioni A, Yuen K, Wang Y (2018). TGFβ attenuates tumour response to PD-L1 blockade by contributing to exclusion of T cells. Nature.

[81] Serrels A, Lund T, Serrels B, Byron A, McPherson RC, von Kriegsheim A (2015). Nuclear FAK controls chemokine transcription, tregs, and evasion of anti-tumor immunity. Cell.

[82] He F, Tay AHM, Calandigary A, Malki E, Suzuki S, Liu T (2023). FPR2 shapes an immune-excluded pancreatic tumor microenvironment and drives T-cell exhaustion in a sex-dependent manner. Cancer Res.

[83] Jaffee EM, Hruban RH, Biedrzycki B, Laheru D, Schepers K, Sauter PR (2001). Novel allogeneic granulocyte-macrophage colony-stimulating factor-secreting tumor vaccine for pancreatic cancer: a phase I trial of safety and immune activation. J Clin Oncol : off j of American Soc of Clin Oncology.

[84] Lutz E, Yeo CJ, Lillemoe KD, Biedrzycki B, Kobrin B, Herman J (2011). A lethally irradiated allogeneic granulocyte-macrophage colony stimulating factor-secreting tumor vaccine for pancreatic adenocarcinoma. A phase II trial of safety, efficacy, and immune activation. Ann Surg.

[85] Laheru D, Lutz E, Burke J, Biedrzycki B, Solt S, Onners B (2008). Allogeneic granulocyte macrophage colony-stimulating factor-secreting tumor immunotherapy alone or in sequence with cyclophosphamide for metastatic pancreatic cancer: a pilot study of safety, feasibility, and immune activation. Clin Cancer Res : an off j of American Assoc Cancer Res.

[86] Lutz ER, Wu AA, Bigelow E, Sharma R, Mo G, Soares K (2014). Immunotherapy converts nonimmunogenic pancreatic tumors into immunogenic foci of immune regulation. Cancer Immunol Res.

[87] Bernhardt SL, Gjertsen MK, Trachsel S, Møller M, Eriksen JA, Meo M (2006). Telomerase peptide vaccination of patients with non-resectable pancreatic cancer: a dose escalating phase I/II study. Br J Cancer.

[88] Mayanagi S, Kitago M, Sakurai T, Matsuda T, Fujita T, Higuchi H (2015). Phase I pilot study of Wilms tumor gene 1 peptide-pulsed dendritic cell vaccination combined with gemcitabine in pancreatic cancer. Cancer Sci.

[89] Middleton G, Silcocks P, Cox T, Valle J, Wadsley J, Propper D (2014). Gemcitabine and capecitabine with or without telomerase peptide vaccine GV1001 in patients with locally advanced or metastatic pancreatic cancer (TeloVac): an open-label, randomised, phase 3 trial. Lancet Oncol.

[90] Ducreux M, Seufferlein T, Van Laethem JL, Laurent-Puig P, Smolenschi C, Malka D (2019). Systemic treatment of pancreatic cancer revisited. Semin Oncol.

[91] Sadelain M, Brentjens R, Rivière I (2013). The basic principles of chimeric antigen receptor design. Cancer Discov.

[92] Sadelain M (2015). CAR therapy: the CD19 paradigm. J Clin Investig.

[93] Brentjens RJ, Rivière I, Park JH, Davila ML, Wang X, Stefanski J (2011). Safety and persistence of adoptively transferred autologous CD19-targeted T cells in patients with relapsed or chemotherapy refractory B-cell leukemias. Blood.

[94] Tsuchikawa T, Takeuchi S, Nakamura T, Shichinohe T, Hirano S (2016). Clinical impact of chemotherapy to improve tumor microenvironment of pancreatic cancer. World J Gastrointest Oncol.

[95] Jackson HJ, Rafiq S, Brentjens RJ (2016). Driving CAR T-cells forward. Nat Rev Clin Oncol.

[96] Sterner RC, Sterner RM (2021). CAR-T cell therapy: current limitations and potential strategies. Blood Cancer J.

[97] Batchu RB, Gruzdyn OV, Mahmud EM, Chukr F, Dachepalli R, Manmari SK (2018). Inhibition of Interleukin-10 in the tumor microenvironment can restore mesothelin chimeric antigen receptor T cell activity in pancreatic cancer in vitro. Surgery.

[98] Pu N, Zhao G, Gao S, Cui Y, Xu Y, Lv Y (2018). Neutralizing TGF-β promotes anti-tumor immunity of dendritic cells against pancreatic cancer by regulating T lymphocytes. Cent Eur J Immunol.

[99] Mace TA, Shakya R, Pitarresi JR, Swanson B, McQuinn CW, Loftus S (2018). IL-6 and PD-L1 antibody blockade combination therapy reduces tumour progression in murine models of pancreatic cancer. Gut.

[100] Zhao X, Subramanian S (2017). Intrinsic resistance of solid tumors to immune checkpoint blockade therapy. Cancer Res.

[101] Vennin C, Murphy KJ, Morton JP, Cox TR, Pajic M, Timpson P (2018). Reshaping the tumor stroma for treatment of pancreatic cancer. Gastroenterology.

[102] Knudsen ES, Vail P, Balaji U, Ngo H, Botros IW, Makarov V (2017). Stratification of pancreatic ductal adenocarcinoma: combinatorial genetic, stromal, and immunologic markers. Clin Cancer Res : an off j of American Assoc Cancer Res.

[103] Liu X, Jia Y, Stoopler MB, Shen Y, Cheng H, Chen J (2016). Next-generation sequencing of pulmonary sarcomatoid carcinoma reveals high frequency of actionable MET gene mutations. J Clin Oncol : off j of American Society Clin Oncology.

[104] Galon J, Mlecnik B, Bindea G, Angell HK, Berger A, Lagorce C (2014). Towards the introduction of the ‘immunoscore’ in the classification of malignant tumours. J Pathol.

[105] Aroldi F, Zaniboni A (2017). Immunotherapy for pancreatic cancer: present and future. Immunotherapy.

[106] Young K, Hughes DJ, Cunningham D, Starling N (2018). Immunotherapy and pancreatic cancer: unique challenges and potential opportunities. Ther Adv Med Oncol.

[107] Morrison AH, Byrne KT, Vonderheide RH (2018). Immunotherapy and prevention of pancreatic cancer. Trends Cancer.

[108] Atkins MB, Lee SJ, Chmielowski B, Tarhini AA, Cohen GI, Truong TG (2023). Combination dabrafenib and trametinib versus combination nivolumab and ipilimumab for patients with advanced BRAF-mutant melanoma: the DREAMseq Trial-ECOG-ACRIN EA6134. J Clin Oncol : off j of American Society Clin Oncology.

[109] Larkin J, Chiarion-Sileni V, Gonzalez R, Grob JJ, Rutkowski P, Lao CD (2019). Five-year survival with combined nivolumab and ipilimumab in advanced melanoma. N Engl J Med.

[110] Tawbi HA, Forsyth PA, Algazi A, Hamid O, Hodi FS, Moschos SJ (2018). Combined nivolumab and ipilimumab in melanoma metastatic to the brain. N Engl J Med.

[111] Wolchok JD, Chiarion-Sileni V, Gonzalez R, Grob JJ, Rutkowski P, Lao CD (2022). Long-term outcomes with nivolumab plus ipilimumab or nivolumab alone versus ipilimumab in patients with advanced melanoma. J Clin Oncol : off j of American Society of Clin Oncology.

[112] Hilmi M, Bartholin L, Neuzillet C (2018). Immune therapies in pancreatic ductal adenocarcinoma: where are we now?. World J Gastroenterol.

[113] Galon J, Bruni D (2019). Approaches to treat immune hot, altered and cold tumours with combination immunotherapies. Nat Rev Drug Discov.

[114] Rojas LA, Sethna Z, Soares KC, Olcese C, Pang N, Patterson E (2023). Personalized RNA neoantigen vaccines stimulate T cells in pancreatic cancer. Nature.

[115] Pant S, Wainberg ZA, Weekes CD, Furqan M, Kasi PM, Devoe CE (2024). Lymph-node-targeted, mKRAS-specific amphiphile vaccine in pancreatic and colorectal cancer: the phase 1 AMPLIFY-201 trial. Nat Med.

[116] Tsujikawa T, Crocenzi T, Durham JN, Sugar EA, Wu AA, Onners B (2020). Evaluation of Cyclophosphamide/GVAX pancreas followed by listeria-mesothelin (CRS-207) with or without nivolumab in patients with pancreatic cancer. Clin Cancer Res : an off j of American Assoc Cancer Resea.

[117] Heumann T, Judkins C, Li K, Lim SJ, Hoare J, Parkinson R (2023). A platform trial of neoadjuvant and adjuvant antitumor vaccination alone or in combination with PD-1 antagonist and CD137 agonist antibodies in patients with resectable pancreatic adenocarcinoma. Nat Commun.

[118] Chen IM, Johansen JS, Theile S, Hjaltelin JX, Novitski SI, Brunak S (2022). Randomized phase II study of nivolumab with or without ipilimumab combined with stereotactic body radiotherapy for refractory metastatic pancreatic cancer (CheckPAC). J Clin Oncol : off j of American Soc Clin Oncology.

[119] Wang Y, Xie T, Xiang S, Liu C, Cheng S, Zhang B (2025). Comparison of immune checkpoint inhibitors in combination with chemotherapy versus chemotherapy alone in the first-line treatment of advanced gastric cancer patients with low PD-L1 expression: a systematic review and meta-analysis. Ther Adv Med Oncol.

[120] Zhao K, Muralidharan V, Brown S, Upton A, Alshimirti M, Cooray PD (2025). Neoadjuvant pembrolizumab enables successful downstaging and resection of borderline resectable MSI-H/dMMR pancreatic ductal adenocarcinoma: a case report and literature review. J Gastrointest Cancer.

[121] Al-Khinji A, Al-Korbi N, Al-Kuwari S, Al-Hor A, Malouche D (2025). Immune checkpoint inhibitors in pancreatic adenocarcinoma: a systematic review and meta analysis of clinical outcomes. Front Oncol.

